# Protein Kinase Signaling Pathways in Plant-*Colletotrichum* Interaction

**DOI:** 10.3389/fpls.2021.829645

**Published:** 2022-01-20

**Authors:** Lingyan Jiang, Shizi Zhang, Jianbin Su, Scott C. Peck, Lijuan Luo

**Affiliations:** ^1^Hainan Key Laboratory for Sustainable Utilization of Tropical Bioresource, College of Tropical Crops, Hainan University, Haikou, China; ^2^Division of Plant Sciences, Interdisciplinary Plant Group, Christopher S. Bond Life Sciences Center, University of Missouri, Columbia, MO, United States; ^3^Division of Biochemistry, Interdisciplinary Plant Group, Christopher S. Bond Life Sciences Center, University of Missouri, Columbia, MO, United States

**Keywords:** protein phosphorylation, plant immunity, fungal virulence, *Colletotrichum* spp., anthracnose

## Abstract

Anthracnose is a fungal disease caused by members of *Colletotrichum* that affect a wide range of crop plants. Strategies to improve crop resistance are needed to reduce the yield losses; and one strategy is to manipulate protein kinases that catalyze reversible phosphorylation of proteins regulating both plant immune responses and fungal pathogenesis. Hence, in this review, we present a summary of the current knowledge of protein kinase signaling pathways in plant-*Colletotrichum* interaction as well as the relation to a more general understanding of protein kinases that contribute to plant immunity and pathogen virulence. We highlight the potential of combining genomic resources and phosphoproteomics research to unravel the key molecular components of plant-*Colletotrichum* interactions. Understanding the molecular interactions between plants and *Colletotrichum* would not only facilitate molecular breeding of resistant cultivars but also help the development of novel strategies for controlling the anthracnose disease.

## Introduction

Protein kinases catalyze reversible phosphorylation, thereby modulating protein activity, conformation, localization, stability and the interacting partners (Cohen, 2000). Multiple protein kinase pathways often coordinate signaling networks and integrate a variety of external and internal cues to regulate the key processes of cellular responses. A large body of evidence demonstrates that phosphorylation is essential for immune responses in plants. In *Arabidopsis*, more than 1,170 phosphopeptides from 472 phosphoproteins were identified after treatments with flg22 or xylanase, which are molecules that could trigger immune responses, suggesting a large amount of phosphorylation events occur during plant immune responses (Benschop et al., 2007; Nühse et al., 2007). In addition, many phosphorylated proteins are key signal transduction components of defense responses, such as receptor-like kinases (RLKs), mitogen activated protein kinases (MAPKs) and calcium-dependent protein kinases (CDPKs) (Couto and Zipfel, 2016; Zhang et al., 2018; Yip Delormel and Boudsocq, 2019).

Fungal pathogens cause severe diseases on a wide range of crops, resulting in significant losses to the economy and agriculture. Although different fungal pathogens have evolved diverse infection and nutrient acquisition strategies, the underlying mechanisms are remarkably conserved; and the phosphorylation/dephosphorylation cycles are essential for conducting these signaling events in fungi. Many fungal protein kinases have been demonstrated to play pivotal roles in development of infection-related structures and for virulence in plant host, and these key protein kinases include the components of cyclic adenosine monophosphate (cAMP)-dependent protein kinases and MAPKs (Turra et al., 2014).

*Colletotrichum* species are among the most economically and scientifically important fungal plant pathogens. The proposed number of species in the *Colletotrichum* genus ranges from 29 to over 700, depending on taxonomic interpretation (Dean et al., 2012). *Colletotrichum* spp. cause anthracnose disease on many crops, including fruits, vegetables, ornamentals, and staple foods in the tropics and subtropics (Dean et al., 2012). Due to the fact that several *Colletotrichum* species could be associated with a single host, disease management is very difficult. Also, a single *Colletotrichum* species could also infect multiple hosts, making anthracnose a severe disease at both preharvest and postharvest stages (Silva et al., 2020). Moreover, *Colletotrichum* can be axenically cultured and genetically manipulated (O'Connell et al., 2004), making it suitable to serve as a model system for hemibiotrophic pathogens.

In this review, we present an overview of the known protein kinase pathways in plant-*Colletotrichum* interactions. This article summarizes the protein kinases associated with the resistance against *Colletotrichum* pathogens in host plants. Meanwhile, we also summarize the protein kinases that govern the infection-related morphogenesis and pathogenesis in *Colletotrichum*. Furthermore, we highlight the potential application of omics techniques to facilitate the understanding of the interaction process and discovery of novel components in the pathways and suggest future directions to improve the breeding of resistant cultivars.

## Protein Kinases in Host Plants

Plants perceive invading pathogens through the recognition of conserved molecular patterns by receptor proteins at plasma membrane, then initiating a series of signaling events, including reactive oxygen species (ROS) production, cellular Ca^2+^ influx, reversible protein phosphorylation, and transcriptional reprogramming (Boller and Felix, 2009). Such basal level resistance is called pattern-triggered immunity (PTI) and plays important roles to ward off most microbes (Jones and Dangl, 2006). To continue a successful invasion in plants, some adapted pathogens have evolved effector proteins that target the key components of PTI and suppress plant resistance. To overcome effector-triggered susceptibility (ETS), plants have further developed intracellular receptor proteins called resistance (R) proteins to monitor effector proteins or their activity and induce effector-triggered immunity (ETI) (Dodds and Rathjen, 2010). During both PTI and ETI, a variety of protein phosphorylation events mediated by protein kinases are important for perception and transduction of signals. Many functional studies and omics analyses have revealed the importance of protein kinases in regulating the plant immunity against *Colletotrichum* pathogens (Tables 1, 2).

**Table 1:** Summary of kinases of plant host associated with resistance.

**Kinase**	**Class**	**Pathosystem**	**Research**	**Reference**
BAK1	RLK	*Arabidopsis*-*C. higginsianum*	Genetic analysis, biochemical analysis	Yamada et al., 2016; Irieda et al., 2019
BIK1	RLCK	*Arabidopsis*-*C. higginsianum*	Genetic analysis, biochemical analysis	Yamada et al., 2016
PEPR1/2	RLK	*Arabidopsis*-*C. higginsianum*	Genetic analysis, biochemical analysis	Yamada et al., 2016
CaRLK family	RLK	Hot pepper-*C. truncatum*	*In silico* analysis, expression analysis	Srideepthi et al., 2020
COK-4-3	RLK	Common bean-*C. lindemuthianum*	*In silico* analysis, expression analysis	Oblessuc et al., 2015
FER-like	RLK	Common bean-*C. lindemuthianum*	*In silico* analysis, expression analysis	Oblessuc et al., 2015
KTR2/3	RLK^a^	Common bean-*C. lindemuthianum*	*In silico* analysis, population genetics, functional analysis	Richard et al., 2014, 2021
MPK3	MAPK	*Arabidopsis*-*C. gloeosporioides*	Genetic analysis, expression analysis of marker genes	Gao et al., 2021
MPK6	MAPK	*Arabidopsis*-*C. gloeosporioides*	Genetic analysis, expression analysis of marker genes	Gao et al., 2021
CDPK5/6/11	CDPK	*Arabidopsis*-*C. gloeosporioides*	Genetic analysis, expression analysis of marker genes	Gao et al., 2021
SIPK	MAPK	*N. benthamiana*-*C. orbiculare*	Genetic analysis, biochemical analysis, subcellular localization	Tanaka et al., 2009; Ishihama et al., 2011
WIPK	MAPK	*N. benthamiana*-*C. orbiculare*	Genetic analysis, biochemical analysis, subcellular localization	Tanaka et al., 2009; Ishihama et al., 2011
NTF4	MAPK	*N. benthamiana*-*C. orbiculare*	Genetic analysis, biochemical analysis, subcellular localization	Tanaka et al., 2009; Ishihama et al., 2011
MEK2	MAPKK	*N. benthamiana*-*C. orbiculare*	Genetic analysis, biochemical analysis	Tanaka et al., 2009; Ishihama et al., 2011
MdMKK4	MAPKK	Apple-*C. fructicola*	Genetic analysis, expression analysis, biochemical analysis, subcellular localization	Shan et al., 2021
MdMPK3	MAPK	Apple-*C. fructicola*	Genetic analysis, expression analysis, biochemical analysis, subcellular localization	Shan et al., 2021
GhMPK7	MAPK	*N. benthamiana*-*C. nicotianae*	*In silico* analysis, genetic analysis, expression analysis, subcellular localization	Shi et al., 2010
GhMPK16	MAPK	*Arabidopsis*-*C. nicotianae*	*In silico* analysis, genetic analysis, expression analysis, subcellular localization	Shi et al., 2011

a *Truncated and chimeric RLK. RLK, receptor-like kinase; RLCK, receptor-like cytoplasmic kinase; CDPK, calcium-dependent protein kinase; MAPK, mitogen activated protein kinase; MAPKK, MAPK kinase; MAPKKK, MAPK kinase kinase*.

**Table 2:** Summary of omics analyses in plant-*Colletotrichum* interaction.

**Pathosystem**	**Omics analysis**	**Identified protein kinases/pathways**	**Reference**
Avocado-*C. gloeosporioides*	Transcriptomics analysis (454 sequencing); time-course experiments	MAPKs, RLKs and CDPKs	Djami-Tchatchou et al., 2012
Maize-*C. graminicola*	Transcriptomics analysis (RNA-seq); comparison of different tissues	LRR-RLK, LecRK and AUR3	Miranda et al., 2017
Apple-*C. gloeosporioides*	Proteomics analysis (2D-PAGE combined with MALDI/TOF MS); time-course experiments; comparison of resistant and susceptible genotypes	MAPK, CDPK	Rockenbach et al., 2015
Sorghum-*C. sublineolum*	Transcriptomics analysis (RNA-seq); time-course experiments; comparison of resistant and susceptible genotypes	MAPK, RLKs	Fu et al., 2020
Walnut-*C. gloeosporioides*	Transcriptomics (RNA-seq) and proteomics (label-free quantitation) analysis; time-course experiments; comparison of resistant and susceptible genotypes	SnRK1	Fang et al., 2021
Strawberry-*C. gloeosporioides*	Phosphoproteomics analysis (label-free quantitation); comparison of resistant and susceptible genotypes	Plant hormone and carbon fixation pathway	Yu et al., 2019
Banana-*C. musae*	Transcriptomics analysis (RNA-seq); melatonin-treated experiments	MAPKs	Li et al., 2019b
Mango-*C.gloeosporioides*	Proteomics analysis (iTRAQ); BABA-treated experiments	CDPK	Li et al., 2019a
Common vetch-*C. lentis*	Transcriptomics analysis (RNA-seq); AM fungus-treated experiments	MAPKs	Ding et al., 2020

*MAPK, mitogen activated protein kinase; RLK, receptor-like kinase; CDPK, calcium-dependent protein kinase; LRR-RLK, leucine-rich repeat receptor-like kinase; LecRK, lectin receptor kinases; AUR3, serine/threonine protein kinase aurora-3; SnRK1, SNF1-related protein kinase 1*.

### Receptor Kinases: Perception of Extracellular Signals

Perception of extracellular stimuli and transduction of signals across the plasma membrane (PM) is crucial for plants to defend against pathogens. In plants, PM-localized pattern recognition receptors (PRRs) are responsible for sensing conserved microbe-associated molecular patterns (MAMPs) or damage-associated molecular patterns (DAMPs) to initiate a series of immune responses (Saijo et al., 2018). Based on different domain structures, plant PRRs primarily consist of receptor-like kinases (RLKs) and receptor-like proteins (RLPs). An RLK contains a variable ectodomain for ligand binding, a single pass transmembrane domain, and a cytoplasmic kinase domain, whereas an RLP lacks the cytoplasmic kinase domain (Tang et al., 2017). Plant RLKs can be further classified based on the type of ligand-binding ectodomain and the presence or absence of a conserved arginine preceding to aspartate in the kinase domain (Couto and Zipfel, 2016). The well-studied RLKs in *Arabidopsis* include FLAGELLIN SENSING 2 (FLS2), EF-TU RECEPTOR (EFR), and PEP RECEPTORs (PEPRs), which recognize bacterial flagellin, bacterial elongation factor Tu and plant endogenous peptides, respectively (Zipfel et al., 2004, 2006; Huffaker et al., 2006). After perception of MAMPs or DAMPs, PRRs form dynamic complexes with regulatory receptor kinases, which is thought to bring the cytoplasmic kinase domains into the proximity necessary for transphosphorylation (Couto and Zipfel, 2016). For instance, *Arabidopsis* FLS2, EFR and PEPR1/2 all recruit the co-receptor kinase BRI1-ASSOCIATED RECEPTOR KINASE 1 (BAK1) upon ligand perception, which is required for immune activation (Chinchilla et al., 2007; Heese et al., 2007; Krol et al., 2010; Liang and Zhou, 2018). A portion of the RLK superfamily possesses the cytoplasmic kinase domain but lacks both extracellular and transmembrane domains, which is referred to as receptor-like cytoplasmic kinases (RLCKs). RLCKs function in concert with RLKs/RLPs and are localized to plasma membrane through N-myristoylation or palmitoylation (Liang and Zhou, 2018). For instance, BOTRYTIS-INDUCED KINASE 1 (BIK1) associates with FLS2 under resting conditions. Upon flg22 elicitation, BAK1 associates with FLS2 and phosphorylates BIK1. Phosphorylated BIK1 in turn phosphorylates FLS2 and BAK1, then dissociates from the FLS2-BAK1 complex (Lu et al., 2010; Zhang et al., 2010; Couto and Zipfel, 2016) to activate downstream signaling components such as RBOHD, a plasma membrane-localized NADPH oxidase, to trigger the ROS burst (Kadota et al., 2014; Li et al., 2014).

**Figure 1:**
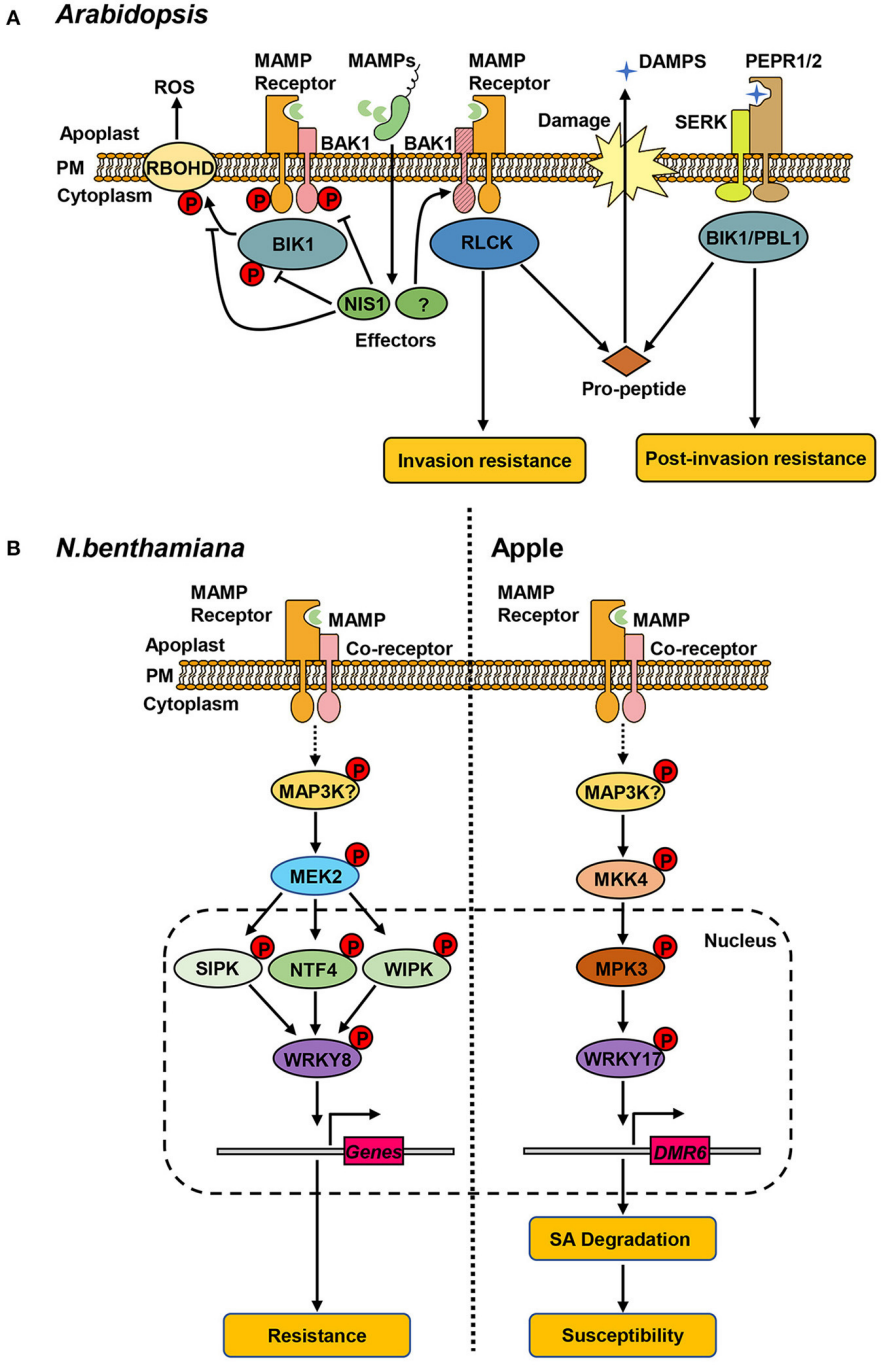
Protein kinase-mediated signaling pathways in plant hosts. **(A)** Receptor kinases associated with resistance against *C. higginsianum* in *Arabidopsis*. Perception of microbe-/damage-associated molecular patterns (MAPMs/DAMPs) by cognate receptors (MAMP receptors, PEPR1/2) leads to the association of co-receptors (BAK1, SERK) and receptor-like cytoplasmic kinases (RLCKs, BIK1), and transphosphorylation within the complex. The phosphorylated BIK1 dissociates from the complex and phosphorylates RBOHD, triggering the production of ROS. Pathogen delivers the effector protein (NIS1) that inhibits the kinase activity of BAK1 and BIK1 and blocks the interaction of BIK1-RBOHD. BAK1 contributes to invasion resistance and is targeted by unknown effector(s) for degradation. BAK1 depletion results in increased release of PROPEP-derived DMAPs, activating the PEPR-mediated pathway that is required for post-invasion resistance. **(B)** MAPK-mediated defense responses in *N. benthamiana* and apple. Left: in *N. benthamiana*, pathogen recognition leads to activation of NtMEK2 by unidentified MAP3K(s). Activated NtMEK2 phosphorylates and activates downstream NtSIPK, NtNTF4, and NtWIPK, which interact with and phosphorylate transcription factor NtWRKY8. NtWRKY8 binds to the promoter of its target genes-related to resistance against *C. orbiculare*. Right: in apple, the MdMEK4-MdMPK3 cascade phosphorylates MdWRKY17 in response to *C. fructicola*. The phosphorylation of MdWRKY17 increases its binding to the promoter of MdDMR6, which promotes SA degradation and increases susceptibility to *C. fructicola*.

In the context of *Arabidopsis*-*C. higginsianum* interactions, BAK1 and BIK1 have been shown to be required for resistance. Quantitative assays of lesion sizes and fungal entry rates revealed that *C. higginsianum* displayed enhanced virulence on *bak1-5, bik1* and *bik1 pbl1* mutants (PBL1 shares partially functional redundancy with BIK1) (Irieda et al., 2019). As they are critical for immunity, BAK1 and BIK1 are targeted by a highly conserved fungal effector, necrosis-inducing secreted protein 1 (NIS1) (Irieda et al., 2019). NIS1 directly interacts with the cytoplasmic region of BAK1 and BIK1, thereby inhibiting their kinase activities. The interaction between NIS1 and BIK1 also blocks the BIK1-RBOHD interaction, thus suppressing PAMP-triggered ROS burst (Irieda et al., 2019). In addition to PTI, BIK1 also contributes to the PEPR-mediated defense against *C. higginsianum* (Yamada et al., 2016). Interestingly, BAK1 is depleted during *C. higginsianum* infection, while PEPR1 accumulates. Consistently, enhanced Pep response and resistance against *C. higginsianum* was observed in *bak1* knockout mutant (Yamada et al., 2016). These results suggest that the PEPR-mediated DAMP signaling ensures the basal resistance when PTI is compromised by BAK1 depletion, revealing a mechanism by which the interaction of MAMP receptors regulate host resistance when PTI is suppressed by virulence effectors (Figure 1A).

Due to the large genome size and difficulties in genetic transformation of crop plants, plant-*Colletotrichum* interactions in non-model plants are not extensively studied. *Co-x* has been identified to be a resistance gene of common bean (*Phaseolus vulgaris*), which confers total resistance to the extremely virulent *C. lindemuthianum* strain 100 (Richard et al., 2014, 2021). The resistance trait segregates as a single dominant gene in the recombinant inbred lines derived from the cross from JaloEEP558 (resistant) and BAT93 (susceptible) (Richard et al., 2014). Sequence comparison of *Co-x* loci between JaloEEP558 and BAT93 revealed that JaloEEP558 contained a unique gene referred to as *PvKTR2/3*, indicating *PvKTR2/3* is a strong candidate gene for *C. lindemuthianum* resistance (Richard et al., 2021). Further studies showed that the presence of *PvKTR2/3* is strictly correlated with resistance in a diverse panel composed of 192 cultivated and wild common beans; and *PvKTR2/3* is induced in response to pathogen inoculation (Richard et al., 2021). Moreover, transient expression of *PvKTR2/3* in a susceptible genotype confirmed the role for *KTR2/3* in *C. lindemuthianum* resistance (Richard et al., 2021). Notably, *PvKTR2/3* encodes a truncated and chimeric CRINKLY4 RLK, which lacks the extracellular and transmembrane domain, suggesting that PvKTR2/3 could be a decoy that mimics a virulence target. Furthermore, PvKTR2/3 lacks the residues required for catalytic activity, suggesting that PvKTR2/3 is a non-functional kinase, which agrees with the hypothesis that PvKTR2/3 is a decoy. Together, these studies showed that *Co-x* is a non-canonical resistance gene, which is interesting for both agronomy and basic research. To further test the decoy model, it would be necessary to generate *PvKRT2/3* loss-of-function mutants by CRISPR and to identify the guard R protein of PvKRT2/3.

In hot pepper (*Capsicum annuum*), 26 non-arginine-aspartate (non-RD) RLKs were identified by an *in silico* approach. By analyzing their expression patterns in both resistant and susceptible cultivars, *CaRLK1, CaRLK15, CaRLK16* were found as candidate genes for resistance against *C. truncatum* (Srideepthi et al., 2020). A similar study has also been performed in common bean, which identified an anthracnose-resistant locus *Co-4* by utilizing the genome information of *Phaseolus vulgaris* and molecular markers (Oblessuc et al., 2015). The *Co-4* locus encodes 24 putative RLKs; and two of them, *PvCOK-4-3* and *PvFER-like*, are responsive to PAMP treatment and pathogen infection (Oblessuc et al., 2015). These studies take advantage of genome data and experimental validation to narrow down candidate RLKs that might contribute to the PAMP signaling and resistance. Precision targeting these candidates is still required to confirm of their roles.

### Mitogen Activated Protein Kinases: From Extracellular Signals to Intracellular Immune Responses

Mitogen-activated protein kinase (MAPK) cascades are highly conserved signaling modules that play important roles in plant growth and defense (Zhang et al., 2018). The cascade is minimally composed of a MAPKKK (MAPK kinase kinase) or MEK kinase (MEKK), a MAPKK (MAPK kinase) or MAPK and ERK kinase (MEK), and a MAPK, linking the signals from upstream RLKs, RLPs or RLCKs to downstream targets via a phosphorylation relay (Pitzschke et al., 2009). The MAPK is the final player in the cascade and is activated by phosphorylation of the conserved Thr and Tyr residues at the activation loop (Meng and Zhang, 2013). Based on sequence similarities, plant MAPKs can be classified into four groups (A, B, C and D). MAPKs in group A, B and C contain a Thr-Glu-Tyr (TEY) activation motif, whereas group D have a Thr-Asp-Tyr (TDY) motif (Meng and Zhang, 2013).

Two *Arabidopsis* MAPKs in group A (AtMPK3 and AtMPK6) were identified to be involved in disease resistance against *C. gloeosporioides* (Gao et al., 2021). Genetic studies have shown that *mpk3-1, mpk6-2* mutants are more susceptible to both weak and aggressive isolates of *C. gloeosporioides*. In addition, marker genes of MAPK pathways, *AtWRKY33* and *AtWRKY40*, are significantly up-regulated in response to *C. gloeosporioides* pathogens, and the less virulent isolates trigger even higher induction of marker genes (Gao et al., 2021). These results suggest a positive role of AtMPK3/6 in regulating resistance against *Colletotrichum*. Upstream kinases and downstream targets of AtMPK3/6 remain to be identified for revealing the resistance mechanisms.

The roles of AtMPK3/6 orthologs in *Nicotiana benthamiana* and apple (*Malus domestica Borkh*.) have been more thoroughly studied. In *Nicotiana benthamiana*, wound-induced protein kinase (NtWIPK) and salicylic acid-induced protein kinase (NtSIPK) have been reported to participate in PTI against the fungal pathogen *C. orbiculare* (Tanaka et al., 2009). Studies have shown that knocking out *CoSSD1* gene reduces the pathogenicity of *C. orbiculare* fungi on *N. benthamiana*. Interestingly, silencing of MAP Kinase Kinase2 (NtMEK2, the common upstream kinase of NtWIPK and NtSIPK) or simultaneously silencing of both NtWIPK and NtSIPK could allow the full infection of the *CoSSD1* knockout mutant pathogen (Tanaka et al., 2009). These results indicate that NtMEK2-NtWIPK/NtSIPK module-mediated defenses contribute to the basal resistance restricting the infection of *C. orbiculare*. In addition, studies have identified the transcription factor NtWRKY8 as the substrate of NtWIPK, NtSIPK and NtNTF4 (a MAPK that shares functionally redundancy with NtSIPK in defense) (Ren et al., 2006; Ishihama et al., 2011). NtWRKY8 can be phosphorylated by all three MAPKs both *in vitro* and *in vivo*, and the phosphorylation increases the DNA binding capacity of NtWRKY8 to its target genes (Ishihama et al., 2011). Ectopic expression of NtWRKY8 phospho-mimicking mutant induced defense-related genes, whereas silencing of NtWRKY8 decreased the expression of defense genes and resistance to pathogen *C. orbiculare* (Ishihama et al., 2011). These results indicate that MAPK-mediated phosphorylation of NtWRKY8 contributes to resistance through activation of downstream defense genes. However, the direct downstream target genes and specific pathways mediated by NtWRKY8 remain to be identified.

In contrast to the results in *Arabidopsis* and *N. benthamiana*, MPK3 in apple (MdMPK3) has been reported to mediate a pathway leading to the susceptibility to *C. fructicola* (Shan et al., 2021). It is shown that MdMPK3 interacts with upstream kinase MdMKK4 on the plasma membrane and in the nucleus, and interacts with downstream transcription factor MdWRKY17 in the nucleus (Shan et al., 2021). *In vitro* kinase assays have shown that the MdMKK4-MdMPK3 cascade phosphorylates MdWRKY17; and the phosphorylation of MdWRKY17 regulates the activation of its target gene *Downy Mildew Resistant 6 (MdDMR6)*, which contributes to the degradation of SA (Shan et al., 2021). Interestingly, the expression levels of MdWRKY17 are significantly higher in six susceptible germplasms compared to six tolerant germplasms post infection, which correlates well with lower SA accumulation (Shan et al., 2021). Therefore, the MdMKK4-MdMPK3-MdWRKY17-MdDMR6 pathway reduces the SA levels and increases the susceptibility to *C. fructicola* (Figure 1B). It will be interesting to further investigate the promoter sequences of MdWRKY17 in different germplasms to identify the SNPs or other variations contributing to different expression levels of MdWRKY17. Compared to the positive roles in *Arabidopsis* and *N. benthamiana*, the differential contribution of MKK4-MPK3 cascades in distinct pathosystems might be resulted from the differentiation of downstream targets.

Several primary studies in cotton (*Gossypium hirsutum*) have indicated positive roles of MAPKs in regulating resistance. *In silico* and localization analysis revealed that GhMPK7 and GhMPK16 are localized in the nucleus, and the expression of both MAPKs are induced in response to pathogen infection (Shi et al., 2010, 2011). Overexpression of GhMPK7 in *N. benthamiana* and GhMPK16 in *Arabidopsis* enhanced the resistance against *C. nicotianae*, which was associated with the increased induction of SA- and ROS-related genes (Shi et al., 2010, 2011). As GhMPK7 and GhMPK16 were localized in nucleus, they might regulate the activity of transcription factors through phosphorylation. In addition, GhMPK7 and GhMPK16 seem to function redundantly based on current studies. It will be interesting to see if they share any functional redundancy by performing the genetic studies using CRISPR system in cotton.

### Omics-Aided Analyses Implicate the Roles of Protein Kinases in Defense Responses

Advances in genomics, transcriptomics, proteomics and metabolomics studies have facilitated the elucidation of cellular processes during host-pathogen interactions. Following sequencing of the reference genome of the hosts and members of *Colletotrichum* spp., many omics-aided studies have inferred the roles of protein kinases in the defense responses against pathogen (Table 2). For instance, the transcriptomes of unharvested and harvested avocado fruit tissues post-inoculation of *C. gloeosporioides* at early stages (1, 4 and 24 h post inoculation) and late stages (3, 4, 7 days post inoculation) were analyzed. The results showed that MAPK and leucine-rich repeat (LRR) RLKs were expressed in all infected samples, whereas CDPKs were expressed in both unharvested and harvested samples during early stages but only in the harvested samples during late stages, suggesting that different protein kinases may function at different stages of pathogen infection (Djami-Tchatchou et al., 2012). In the study of maize-*C. graminicola* interaction, changes in global gene expression were studied in root, male and female inflorescences of maize under local and systemic fungal infection treatments. The results revealed that LRR RLKs, lectin receptor kinases (LecRKs) and serine/threonine protein kinase aurora-3 (AUR3) were significantly induced in the roots during local infection (Miranda et al., 2017).

In addition to time-course analyses, comparative studies of resistant and susceptible genotypes also indicate that protein kinases might be involved in regulating the resistance against pathogens. Proteomics approaches based on 2-dimensional polyacrylamide gel combined with MALDI/TOF mass spectrometry were applied to study the differentially expressed proteins of apple cv. Fuji (resistant) and cv. Gala (susceptible) in response to *C. gloeosporioides* infection. The analysis showed that a MAPK protein was uniquely expressed in cv. Gala, whereas a CDPK was in cv. Fuji (Rockenbach et al., 2015). Genome-wide mRNA and microRNA profiles of resistant and susceptible sorghum genotypes showed that genes encoding RLKs and MAPKs were specifically induced in the resistant genotype (Fu et al., 2020). Phosphoproteomics analysis of resistant and susceptible strawberry cultivars in response to *C. gloeosporioides* identified two specific phosphorylation motifs of differentially expressed phosphopeptides in resistant cultivars, and the phosphorylated peptides were highly enriched in the plant hormone signaling and carbon fixation pathways (Yu et al., 2019). The co-expression network analysis of transcriptomic profiles in resistant and susceptible walnut fruit bracts infected by *C. gloeosporioides* at various lifestyles identified nine hub genes, and one of them encodes the α-subunit of SNF1-related protein kinase 1 (SnRK1) (Fang et al., 2021).

MAPKs and CDPKs have also been implicated in the induced resistance against *C. musae, C. gloeosporioides* and *C. lentis*. Banana anthracnose is caused by *C. musae*, and exogenous melatonin treatment could significantly reduce the incidence of anthracnose. Transcriptomic analysis of banana peels showed that the MAPK signaling pathway was significantly enhanced after melatonin treatment, which might be involved in the enhanced fruit resistance (Li et al., 2019b). β-aminobutryric acid (BABA) is an environmentally friendly agent used to induce resistance by priming of defense in plants. Priming is an important inducible defense mechanism in plants, which could put plants in a standby state and help plants respond more rapidly and efficiently once affected by pathogens (Li et al., 2019a). Investigation of priming mechanism of BABA-induced resistance was performed on mango-*C. gloeosporioides* interaction using iTRAQ-based proteomic approach. The proteomics analysis showed specific upregulation of CDPKs in the fruit treated by BABA post inoculation, which might lead to more rapid and robust fight against pathogen (Li et al., 2019a). The arbuscular mycorrhizal (AM) fungus *Sieverdingia tortuosa* could reduce anthracnose severity of common vetch caused by *C. lentis*. Transcriptomics analysis showed enhanced expression of genes in MAPK signaling pathways in response to AM fungi treatment, suggesting that AM fungi might increase the defense pathways and decrease the severity of anthracnose (Ding et al., 2020).

These omics-aided studies have provided a large pool of potential protein kinases that might regulate the plant resistance against *Colletotrichum* pathogens. The following studies could focus on demonstrating their functions by genetic studies and biochemical analysis.

## Protein Kinases in *Colletotrichum*

**Figure 2:**
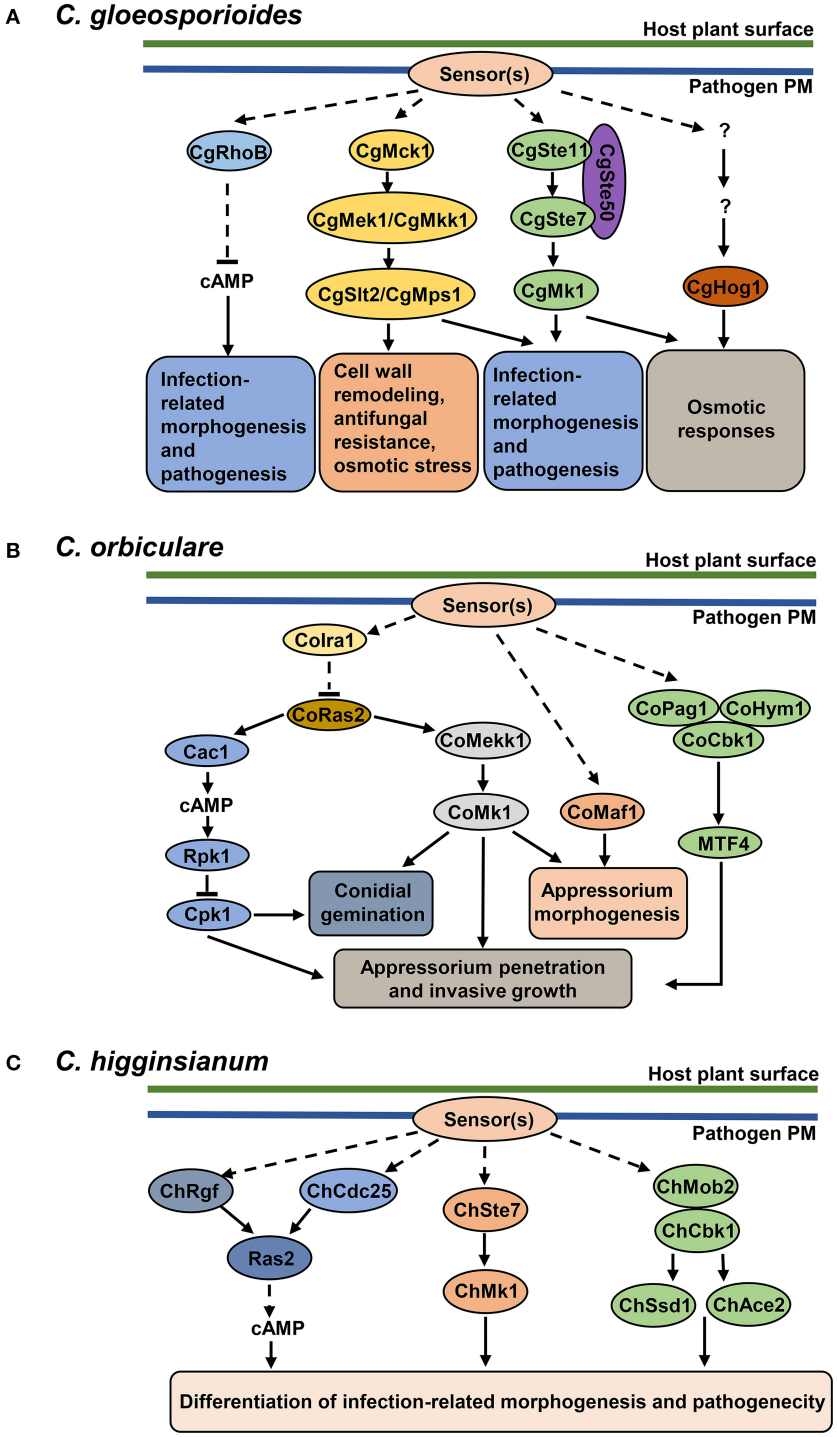
Protein kinase pathways in *Colletotrichum* pathogens. **(B)** cAMP signaling pathway, MAPK cascades and MOR pathway in *C. orbiculare*. cAMP-PKA signaling pathway (adenylate cyclase Cac1, PKA regulatory unit Rpk1, PKA catalytic unit Cpk1) is involved in conidial germination, appressorium penetration and invasive growth. The CoMekk1-CoMk1 module is involved in conidial germination, appressorium formation and penetration. CoMaf1 is involved in appressorium formation. The MOR pathway (scaffold proteins CoPag1 and CoHym1, NDR kinase CoCbk1 and transcription factor MTF4) is involved in appressorium penetration and invasive growth. GTPase-activating protein CoIra1 and GTPase CoRas2 function upstream of cAMP and MAPK signaling pathways. CoIra1 inhibits the activity of CoRas2, and CoRas2 positively regulates cAMP and MAPK pathways. **(A)** cAMP signaling pathway and MAPK cascades in *C. gloeosporioides*. GTPase protein RhoB downregulates cAMP levels and controls the development of infection-related structures and pathogenicity. CgMck1-CgMek1/CgMkk1-CgSlt2/CgMps1 and CgSte11-CgSte7-CgMk1 cascade regulate infection-related morphogenesis and pathogenesis. CgMck1-CgMek1/CgMkk1-CgSlt2/CgMps1 cascade regulates cell wall remodeling and stress responses. CgSte11-CgSte7-CgMk1 and CgHog1-mediated cascades contribute to osmotic responses. **(C)** cAMP, MAPK and MOR pathway in *C. higginsianum*. All three pathways regulate the differentiation of infection-related morphogenesis and pathogenicity. Guanine exchange factors ChRgf and ChCdc25 activate Ras2 and positively regulate cAMP levels. *C. higginsianum* MAPK pathway is composed of MAPKK ChSte7 and MAPK ChMk1. In MOR pathway, ChMob2 binds to ChCbk1, forming an active complex, which potentially regulates transcription factors ChSsd1 and ChAce2.

Most *Collotrichum* spp. are hemibiotrophic fungal pathogens and develop a set of specialized infection structures in host plants, including germ tubes, appressoria, biotrophic hyphae, and necrotrophic hyphae (O'Connell et al., 2012). In *Collotrichum* spp., reversible protein phosphorylation by protein kinases is involved in the regulation of various growth and developmental processes associated with pathogenesis and responding to plant-surface signals. Key components of the MAPK cascade, cyclic AMP-dependent Protein Kinase A (cAMP-PKA) pathway, morphogenesis-related NDR kinase network (MOR) and two-component system have been reported to play important and conserved roles in the infection of *Colletotrichum* pathogens (Table 3, Figure 2).

**Table 3:** Summary of protein kinases regulating morphogenesis and pathogenesis of *Colletotrichum*.

**Kinase**	**Class**	**Pathway**	**Pathosystem**	**Function**	**Reference**
Ct-PKAC	PKA catalytic subunit	cAMP-PKA signaling	*C. trifolii*-alfalfa	Vegetative growth, conidiation, appressorium formation, pathogenicity	Yang and Dickman, 1999
Co-Rpk1	PKA regulatory subunit	cAMP-PKA signaling	*C. orbiculare*-cucumber	Vegetative growth, conidiation, pathogenicity	Takano et al., 2001
Co-Cpk1	PKA catalytic subunit	cAMP-PKA signaling	*C. orbiculare*-cucumber	Conidial germination, appressorium function, pathogenicity	Yamauchi et al., 2004
Cg-PKAC	PKA catalytic subunit	cAMP-PKA signaling	*C. gloeosporioides*-mango	Conidial germination, appressorium function, pathogenicity	Priyatno et al., 2012
Ch-PKA1	PKA catalytic subunit	cAMP-PKA signaling	*C. higginsianum*-*Arabidopsis*	Hyphal growth, conidiation, appressorium formation, stress responses (cell wall, elevated temperatures and exogenous H2O2), pathogenicity	Zhu et al., 2017
Co-Mk1	MAPK	MAPK signaling	*C. orbiculare*-cucumber	Conidiation, conidial germination, appressorium maturation (on host plants and glass), melanization, pathogenicity	Takano et al., 2000; Kojima et al., 2002
Ct-Pmk1	MAPK	MAPK signaling	*C. truncatum*-soybean	Vegetative growth, conidiation, melanization, appressorium formation, pathogenicity	Xiong et al., 2015
Ch-Mk1	MAPK	MAPK signaling	*C. higginsianum*-*Arabidopsis*	Cell wall integrity, growth rate, conidiation, appressorium formation, melanization, pathogenicity	Wei et al., 2016
Cg-Mk1	MAPK	MAPK signaling	*C. gloeosporioides*-polar	Hyphal growth, appressorium formation, melanization, osmotic stress, pathogenicity	He et al., 2017
Cf-Pmk1	MAPK	MAPK signaling	*C. fructicola*-apple/pear	Growth rate, conidiation, appressorium formation, melanization, pathogenicity	Liang et al., 2019
Cg-Slt2/Mps1	MAPK	MAPK signaling	*C. gloeosporioides*-mango	Conidiation, polarized growth, appressorium formation, pathogenicity	Yong et al., 2013
Co-Maf1	MAPK	MAPK signaling	*C. orbiculare*-cucumber	Conidiation, early differentiation of appressorium formation (on host plants and glass), pathogenicity	Kojima et al., 2002
Cg-Ste11	MAPKKK	MAPK signaling	*C. gloeosporioides*-polar	Appressorium formation, conidial germination, vegetative growth, melanization, ROS accumulation, abiotic stress resistance (nitrogen and osmotic stress), pathogenicity	Wang et al., 2021
Co-Mekk1	MAPKK	MAPK signaling	*C. orbiculare*-cucumber	Conidiation, conidial germiantion, appressorium formation, pathogenicity, osmotic stress	Sakaguchi et al., 2010
Cg-Ste7	MAPKK	MAPK signaling	*C. gloeosporioides*-polar	Appressorium formation, conidial germination, vegetative growth, melanization, ROS accumulation, abiotic stress resistance (nitrogen and osmotic stress), pathogenicity	Wang et al., 2021
Ch-Ste7	MAPKK	MAPK signaling	*C. higginsianum*-*Arabidopsis*	Vegetative growth, melanization, appressorium formation, pathogenicity	Yuan et al., 2016
Cg-Mck1	MAPKKK	MAPK signaling	*C. gloeosporioides*-polar/Chinese fir	Vegetative growth, cell wall integrity, conidiation, appressorium formation, pathogenicity	Fang et al., 2018
Cg-Mek1/Mkk1	MAPKK	MAPK signaling	*C. gloeosporioides*-avacodo	Cell division, germination, and appressorium formation of conidia induced by hard surface contact and host signals	Kim et al., 2000
Cg-Hog1	MAPK	MAPK signaling	*C. gloeosporioides*-polar	Osmotic stress and fludioxonil resistance	Li et al., 2020
Co-Cbk1	NDR	MOR pathway	*C. orbiculare*-cucumber	Appressorium formation, infection-related gene expression triggered by plant-derived signals, pathogenicity	Kodama et al., 2017
Ch-Cbk1	NDR	MOR pathway	*C. higginsianum*-*Arabidopsis*	Conidiation and pathogenicity	Schmidpeter et al., 2017
Cl-Sln1	Histidine kinase	Two component system	*C. lindemuthianum*-common bean	Appressorium formation, melanization, pathogenicity	Nogueira et al., 2019

*PKA, protein kinase A; cAMP, cyclic adenosine monophosphate; MAPK, mitogen activated protein kinase; MAPKK, MAPK kinase; MAPKKK, MAPK kinase kinase; NDR, Dbf2-related protein kinase; MOR, morphogenesis-related NDR kinase*.

### Fungal MAPK Cascades

Fungal MAPK cascades function in succession to transmit a variety of extracellular stimuli to pathogenicity responses (Widmann et al., 1999). The model yeast *Saccharomyces cerevisiae* genome encodes five MAPKs, namely Fus3, Kss1, Slt2, Hog1, and Smk1 (Xu, 2000). However, filamentous fungi usually have three MAPKs that are orthologs of *S. cerevisiae* Fus3/Kss1, Slt2, and Hog1. These three MAPK pathways mediate signaling cascades to regulate infection-related morphogenesis, cell wall remodeling, and high osmolarity response, all contributing to the virulence on plants (Turra et al., 2014).

Fus3/Kss1 orthologs were shown to be essential for plant infection in *Colletotrichum* spp., including CoMk1 in *C. orbiculare* (Takano et al., 2000; Kojima et al., 2002), CtPmk1 in *C. truncatum* (Xiong et al., 2015), ChMk1 in *C. higginsianum* (Wei et al., 2016), CgMk1 in *C. gloeosporioides* (He et al., 2017), and CfPmk1 in *C. fructicola* (Liang et al., 2019). Studies in these *Colletotrichum* species have shown that Fus3/Kss1-related MAPKs are required for both differentiation of penetration structures (conidiation, appressorium formation, hyphal growth and melanization) and pathogenic growth *in planta*, suggesting conserved roles of Fus3/Kss1 MAPKs in regulating the fungal development and pathogenesis (Takano et al., 2000; Kojima et al., 2002; Xiong et al., 2015; Wei et al., 2016; He et al., 2017; Liang et al., 2019). However, the role of Fus3/Kss1 type of MAPKs varies among different *Colletotrichum* species. For instance, the deletion mutant of *CoMk1* in *C. orbiculare* failed to germinate on both host plants and glass surfaces, demonstrating that the CoMk1 regulates conidial germination (Takano et al., 2000). In contrast to CoMk1, the conidia from the *Ctpmk1* mutant of *C. truncatum* germinated normally on glass slides and onion epidermal surfaces (Xiong et al., 2015). Moreover, the deletion mutants of *CtPmk1, ChMk*1, *CgMk*1 and *CfMk1* showed an attenuated growth rate, whereas the mutant of *CoMk1* did not (Xiong et al., 2015; Wei et al., 2016; Liang et al., 2019). In addition to the roles in pathogenesis, some members of the Fus3/Kss1 type MAPK have been shown to participate in responding to high osmosis and cell wall stresses. For instance, the deletion of *CoMk1, CgMk1* and *CfPmk1* resulted in hypersensitivity to high osmotic treatment, and the deletion mutants of *ChMk1* were sensitive to cell wall inhibitors (Sakaguchi et al., 2010; Xiong et al., 2015; Wei et al., 2016; He et al., 2017). Overall, current studies have indicated that Fus3/Kss1 type MAPKs are central regulators of infectious growth in host plants and other stress responses.

Other components of the Fus3/Kss1 MAPK module, such as the upstream MAPKK Ste7, the MAPKKK Ste11, and the adaptor protein Ste50, were also essential for appressorium formation, penetration of the cellophane membrane, invasive growth and pathogenecity in *C. gloeosporioides* (Wang et al., 2021). Furthermore, the study showed that CgSte50, CgSte11, and CgSte7 positively regulate the phosphorylation of CgMk1, which is involved in ROS accumulation during conidial germination and osmotic stress response (Wang et al., 2021) (Figure 2A). The function of the Ste11 ortholog in *C. orbiculare*, CoMEKK1, has been also characterized. The disrupted mutants of *CoMekk1* and *CoMk1* were sensitive to osmotic stress; and the nuclear localization of CoMk1 induced by salt stress was diminished in *Comekk1* mutant, placing CoMekk1 upstream of CoMk1 (Sakaguchi et al., 2010) (Figure 2B). Interestingly, overexpression of constitutively active form of the CoMekk1 in wild type and *Comekk1* mutant showed slower hyphal growth and abnormal appressorium (Sakaguchi et al., 2010). This suggests that precise regulation of MAPK phosphorylation is essential for vegetative growth and appressorium formation. Consistently, the *ChSte7* disruption mutants showed extremely decreased growth rates and defects in appressorium formation in *C. higginsianum*; and the mutants even failed to cause lesions on wounded leaves of *Arabidopsis*, suggesting the function of fungal MAPKs in pathogenicity reaches beyond the differentiation of penetration structures (Yuan et al., 2016) (Figure 2C). Collectively, these studies have offered a body of evidence for a conserved role of the Ste11-Ste7-Fus3/Kss1 MAPK module in different *Colletotrichum* species during invasive growth and infection on plants.

The Slt2 MAPK in *S. cerevisiae* regulates cell wall remodeling during cell cycle and various stress responses by regulating cell wall biosynthesis and actin organization to maintain the cell wall integrity (CWI) (Levin, 2005). The core components of CWI module includes MAPKKK protein Bck1/Mck1, MAPKK protein Mkk1/2 and MAPK protein Slt2/Mps1. Studies have shown that CWI pathway genes are involved to regulate invasive structures development and virulence to plant hosts in *C. gloeosporioides* and *C. orbiculare*. Slt2 orthologs are required for the developmental process of conidiation and appressorium formation. Due to the higher amount of bipolar germination of conidia, appressorium formation was greatly reduced in *C. gloeosporioides slt2* mutants and *C. orbiculare maf1* mutants, and thus reduced in virulence (Kojima et al., 2002; Yong et al., 2013) (Figures 2A,B). Disruption of the upstream MAPKK CgMkk1 and MAPKKK CgMck1 resulted in a similar phenotype as *slt2* mutants (Kim et al., 2000; Fang et al., 2018). In addition, the CgMck1-CgMkk1-CgSlt2 pathway has been shown to regulate the cell wall integrity as the mutants are hypersensitive to cell wall stress, antifungal bacterium agent and osmotic stress (Fang et al., 2018) (Figure 2A). These results suggest that Stl2-mediated cascades might play a protective role for fungi against cell wall-degrading enzymes and against other components of immune responses, such as plant antimicrobial peptides.

The Hog MAPK pathway governs adaptive responses to hyperosmotic stress (Saito and Posas, 2012). Studies in *C. gloeosporioides* showed that the deletion of *CgHog1* resulted in enhanced sensitivity to osmotic stress and increased resistance to fludioxonil. Further transcriptomic profiles of wild type and *Cghog1* mutant in response to sorbitol and fludioxonil indicate that CgHog1 may regulate the adaption to osmotic stress by controlling the synthesis and accumulation of osmolytes; and the growth defect caused by fludioxonil in *Cghog1* mutants may be associated with disruption of endocytosis (Li et al., 2020) (Figure 2A). Although studies in other plant-pathogenic fungi have shown that Hog1 is important for virulence (Turra et al., 2014), its contribution to pathogenicity in *Colletotrichum* species still need to be elucidated.

Molecular mechanisms that modulate the activity of the MAPK pathways during *Colletotrichum* infection have not been extensively characterized. Two GTP-binding proteins, Ras and Rac, function oppositely upstream of the *C. trifolii* MAPK pathway to regulate hyphal growth and development (Truesdell et al., 1999; Chen and Dickman, 2004). A dominant active mutation of Ras (DARas) inhibits MAPK pathway and causes abnormal hyphal morphogenesis (Truesdell et al., 1999). However, co-expression of a dominant active Rac1, DARac, in the DARas mutant background induces MAPK activation and restores the wild-type phenotype. In addition, Ct-Ras directly interacts with Ct-Rac1, and DARas inhibits the expression of Ct-Rac1 (Chen and Dickman, 2004). This suggests that Ct-Rac1 functions downstream of CtRas, and RasRac form a complex to fine-tune the activity of MAPKs. In *C. orbiculare*, expression of DA CoMEKK1 restores the appressorium formation in *Coras2* mutant, but expression of DA CoRas2 in *Comekk1* mutant could not reverse the phenotype, suggesting that CoRas2 is an upstream regulator of the MAPK signaling pathway (Harata and Kubo, 2014). In addition, downstream MAPK CoMk1 is highly phosphorylated by the expression of DA CoRas2, suggesting that CoRas2 positively regulates the phosphorylation of CoMk1 (Harata and Kubo, 2014). However, these studies have not demonstrated whether null or DA alleles of Ras and/or Rac would affect plant infection. Thus, the direct connection of Ras or Rac proteins to MAPK-mediated regulation of pathogenicity still needs to be further investigated.

### Fungal cAMP-PKA Signaling Pathways

The cAMP signaling pathway has been shown to play pivotal roles in fungal pathogenesis (Kronstad et al., 2011). cAMP is produced from ATP by adenylate cyclase (AC) and controls the activity of PKA, which is a tetrameric holoenzyme composed of two regulatory subunits (RPK) and two catalytic subunits (CPK). Binding of cAMP to the regulatory subunits of PKA enables the catalytic subunits to phosphorylate target proteins in the cAMP-regulated processes (Robertson and Fink, 1998).

Molecular characterization of AC, RPK, and CPK have confirmed the crucial role of cAMP-PKA signaling in regulating pathogenesis-related developmental processes. For instance, mutants lacking the catalytic subunit or regulatory subunit of PKA of *C. trifolii, C. gloeosporioide*s, *C. orbiculare*, and *C. higginsianum* all showed defects in morphogenesis of infection structures and reduced pathogenesis in host plants (Yang and Dickman, 1999; Takano et al., 2001; Yamauchi et al., 2004; Priyatno et al., 2012; Zhu et al., 2017) (Table 3, Figure 2).

In *C. gloeosporioides*, intracellular levels of cAMP are modulated by CgRhoB, a Rho-GTPase protein. Disruption of *CgRhoB* results in intracellular cAMP accumulation, causing the lower conidial germination and abnormal appressorium formation. In addition, these mutants also showed defects in cell wall integrity and pathogenicity (Xu et al., 2016) (Figure 2A). *C. higginsianum* mutants lacking guanine exchange factors (GEF), ChRgf or ChCdc25, exhibit reduced intracellular cAMP levels and defects in vegetative growth, conidial germination and virulence, which could be restored by exogenous cAMP treatment (Gu et al., 2017; Yan et al., 2020). Importantly, ChRgf and ChCdc25 may interact with Ras2 and affect Ras2 protein abundance in *C. higginsianum*, suggesting that Ras2 may be a downstream target of ChRgf and ChCdc25 (Gu et al., 2017; Yan et al., 2020) (Figure 2C). Taken together, these results indicate that the precise regulation of cAMP levels is required for proper development of infection-related structure and pathogen virulence. These studies also highlight a regulatory role of RhoB, GEF and Ras proteins upstream of the cAMP-PKA pathway.

Cross-talk between cAMP-PKA pathway and MAPK pathway has been reported in *C. orbiculare*. The Ras GTPase-activating protein CoIra1 and Ras protein CoRas2 orchestrates conidial germination, appressoirum penetration and invasive growth of *C. orbiculare* upstream of cAMP-PKA and Mk1 MAPK pathway. CoIra1 interacts with and negatively regulates CoRas2. CoRas2 positively regulates the cAMP levels and Mk1 activation as evidenced by the increased cAMP level and Mk1 activity in DA mutants (Harata and Kubo, 2014). As CoIra1-CoRas2 regulates the same process through both cAMP-PKA and MAPK pathways, it would be interesting to see if these two pathways control the downstream responses through common or different targets. Collectively, these studies above support a notion that the cAMP-PKA pathway functions as a conserved regulator and cooperates with the MAPK cascade in controlling the fungal development of penetration structure and invasive growth in plant.

### Fungal MOR Pathways

The nuclear Dbf2-related (NDR) protein kinases are core members of the morphogenesis-related NDR kinase pathway (MOR), and control the cell polarity, which is crucial for plant cell invasion, the production of specialized penetration structures, and the spread of the fungi through the host tissues (Hergovich et al., 2006; Turra et al., 2014). In *C. orbiculare*, inhibition of the NDR kinase CoCbk1 results in bilateral germination of conidia, abnormal appressorium morphology, and reduced pathogenicity, suggesting that CoCbk1 activity is essential for morphological differentiation of infection structures and pathogenesis (Kodama et al., 2017). Two scaffold proteins, CoPag1 and CoHym1, directly interact with CoCbk1 and regulate the phosphorylation of CoCbk1 (Kodama et al., 2017). The *C. orbiculare* MOR pathway, CoPag1-CoHym1-CoCbk1, functions in sensing the cutin monomer, n-octadecanal, released from the host cuticle by conidial esterases, thus activating the plant-signal-induced genes to potentially facilitate infection (Kodama et al., 2017). CoCbk1 interacts with a Zn(II)_2_Cys_6_ transcription factor CoMTF4 and regulates its expression. Loss of *CoMTF4* causes phenotypes similar to that of the MOR pathway mutants; and epistasis analysis with DA CoMTF4 confirms that CoMTF4 functions downstream of MOR pathway (Kodama et al., 2017) (Figure 2B). Meanwhile, ChCbk1 and ChMob2 in *C. higginsianum* are also found to be essential for the conidiation, appressorium formation and pathogenicity (Schmidpeter et al., 2017). Deletion of *ChSSD1* and *ChACE2*, two transcription factors, leads to the similar phenotypes as *ChCbk1* mutants, placing them as potential downstream targets of ChCbk1 (Schmidpeter et al., 2017) (Figure 2C). Taken together, these findings highlight a role for MOR pathway in mediating recognition of plant signals and stimulating infection-related infection.

### Fungal Two-Component Phosphorelay System

Histidine kinases (HKs) function at the head of two-component phosphorelay systems (TCSs), a conserved signaling module in both prokaryotes and eukaryotes (Turra et al., 2014). Fungal TCSs consist of an upstream hybrid HK containing an HK domain and a C-terminal response regulator (RR) domain, an intermediate histidine-containing phospho-transfer (HPt) protein, and a downstream RR protein (Li et al., 2010). In *S. cerevisiae* and *M. oryzae*, the HK Sln1 (class VI) has been reported to be required for osmotic stress resistance and function upstream of the Hog1 MAPK pathway. In *Colletotrichum* species, the HK family orthologous to Sln1 has only been examined in *C. lindemuthianum*. ClSln1 contains a N-terminal transmembrane domain, five typical domains of HKs, and a C-terminal RR domain. Loss of *ClSln1* causes a deficiency in the production and melanization of appressoria, as well as complete loss of pathogenicity on bean leaves (Nogueira et al., 2019). It appears that ClSln1 contributes to the virulence mainly by controlling the formation of penetration structure. Future studies on the biological role of ClSln1 could investigate whether ClSln1 function through MAPK pathway like that in *S. cerevisiae* and *M. oryzae* or through other novel pathways in *Colletotrichum* species.

## Conclusions and Perspectives

The plant-*Colletotrichum* interaction is an economically important pathosystem. However, little is known about the molecular aspects of these interaction processes. Understanding the function of key players would facilitate the improvement of the crop's resistance against pathogens in the genus *Colletotrichum*.

In plant hosts, several receptor kinases and MAPK modules have been functionally characterized to be critical for anthracnose resistance. These defense-related genes could either be transferred into elite crop materials for resistance improvement or serve as biomarkers for identification of resistance sources. Advances in sequencing technologies, data processing tools and bioinformatics software make omics studies possible in investigating plant-*Colletotrichum* interactions. Multiple omics-aided studies have indicated roles of proteins kinases in the defense responses and resistance mechanisms; however, functional validation of these protein kinases are largely delayed. Most omics studies so far are limited to the transcriptomics and the proteomics of total proteins, and few phosphoproteomics studies have been performed in plant-*Colletotrichum* pathosystem. Combined with genome information of diverse germplasms, phosphoproteomics could facilitate the discovery of novel components of signaling networks and the evolution of signaling pathways.

In *Colletotrichum* spp., several conserved PK-based signaling pathways have been shown to govern the infection-related morphogenesis and pathogenesis. Thus, targeting these pathways could potentially be a good strategy to control the anthracnose diseases. Although multiple key components of these pathway have been characterized, some important gaps remain to be filled. One important area is the characterization of host-derived signals and understanding sensing mechanisms by fungi. In line with such idea, downstream targets of these pathways are also worth to be identified. Another interesting area for future studies is the elucidation of how signal separation and integration between PK cascades is accomplished. Moreover, the genomic resources and omics studies of *Colletotrichum* spp. could promote the identification of other novel components of pathogen virulence and understanding of their interaction with plant hosts.

## Author Contributions

LJ, SZ, JS, and SP conceived the main subject of this review and wrote the manuscript. LL provided the suggestions and revisions. All authors contributed to the article and approved the submitted version.

## Funding

This work was supported by the National Natural Science Foundation of China (31960342), Young Elite Scientists Sponsorship Program by CAST (Project No. 2020QNRC001), and Young Talents' Science and Technology Innovation Project of Hainan Association for Science and Technology (QCXM202001).

## Conflict of Interest

The authors declare that the research was conducted in the absence of any commercial or financial relationships that could be construed as a potential conflict of interest.

## Publisher's Note

All claims expressed in this article are solely those of the authors and do not necessarily represent those of their affiliated organizations, or those of the publisher, the editors and the reviewers. Any product that may be evaluated in this article, or claim that may be made by its manufacturer, is not guaranteed or endorsed by the publisher.

## References

[B1] Benschop J. J. Mohammed S. O'Flaherty M. Heck A. J. Slijper M. Menke F. L. (2007). Quantitative phosphoproteomics of early elicitor signaling in *Arabidopsis*. Mol. Cell. Proteomics 6, 1198–1214. 10.1074/mcp.M600429-MCP200 17317660

[B2] Boller T. Felix G. (2009). A renaissance of elicitors: perception of microbe-associated molecular patterns and danger signals by pattern-recognition receptors. Ann. Rev. Plant Biol. 60, 379–406. 10.1146/annurev.arplant.57.032905.105346 19400727

[B3] Chen C. Dickman M. B. (2004). Dominant active Rac and dominant negative Rac revert the dominant active Ras phenotype in *Colletotrichum trifolii* by distinct signalling pathways. Mol. Microbiol. 51, 1493–1507. 10.1111/j.1365-2958.2003.03932.x 14982641

[B4] Chinchilla D. Zipfel C. Robatzek S. Kemmerling B. Nürnberger T. Jones J.. (2007). A flagellin-induced complex of the receptor FLS2 and BAK1 initiates plant defence. Nature. 448, 497–500. 10.1038/nature05999 17625569

[B5] Cohen P (2000). The regulation of protein function by multisite phosphorylation–a 25 year update. Trends Biochem. Sci. 25, 596–601. 10.1016/S0968-0004(00)01712-6 11116185

[B6] Couto D. Zipfel C. (2016). Regulation of pattern recognition receptor signalling in plants. Nat. Rev. Immunol. 16, 537–552. 10.1038/nri.2016.77 27477127

[B7] Dean R. Van Kan J. A. Pretorius Z. A. Hammond-Kosack K. E. Di Pietro A. Spanu P. D.. (2012). The top 10 fungal pathogens in molecular plant pathology. Mol. Plant Pathol. 13, 414–430. 10.1111/j.1364-3703.2011.00783.x 22471698 PMC6638784

[B8] Ding T. Zhang W. Li Y. Duan T. (2020). Effect of the AM fungus *Sieverdingia tortuosa* on common vetch responses to an anthracnose pathogen. Front. Microbiol. 11:542623. 10.3389/fmicb.2020.542623 33391193 PMC7775565

[B9] Djami-Tchatchou A. T. Straker C. J. Allie F. (2012). 454 sequencing for the identification of genes differentially expressed in avocado fruit (cv. fuerte) infected by Colletotrichum gloeosporioides. J. Phytopathol. 160, 449–460. 10.1111/j.1439-0434.2012.01925.x

[B10] Dodds P. N. Rathjen J. P. (2010). Plant immunity: towards an integrated view of plant-pathogen interactions. Nat. Rev. Genet. 11, 539–548. 10.1038/nrg2812 20585331

[B11] Fang H. Liu X. Dong Y. Feng S. Zhou R. Wang C.. (2021). Transcriptome and proteome analysis of walnut (*Juglans regia* L.) fruit in response to infection by *Colletotrichum gloeosporioides*. BMC. Plant Biol. 21:249. 10.1186/s12870-021-03042-1 34059002 PMC8166054

[B12] Fang Y. Xia L. Wang P. Zhu L. H. Ye J. Huang L. (2018). The MAPKKK CgMck1 is required for cell wall integrity, appressorium development, and pathogenicity in *Colletotrichum gloeosporioides*. Genes. 9:543. 10.3390/genes9110543 30413120 PMC6267176

[B13] Fu F. Girma G. Mengiste T. (2020). Global mRNA and microRNA expression dynamics in response to anthracnose infection in sorghum. BMC Genomics. 21, 760. 10.1186/s12864-020-07138-0 33143636 PMC7641857

[B14] Gao M. Wan M. Yang L. Zhao M. Liu X. Chen J.. (2021). Molecular and physiological characterization of *Arabidopsis*–*Colletotrichum gloeosporioides* pathosystem. Plant Pathol. 70:1168–1179. 10.1111/ppa.13364

[B15] Gu Q. Chen M. Huang J. Wei Y. Hsiang T. Zheng L. (2017). Multifaceted roles of the Ras guanine-nucleotide exchange factor *ChRgf* in development, pathogenesis, and stress responses of *Colletotrichum higginsianum*. Phytopathology. 107:433–443. 10.1094/PHYTO-03-16-0137-R 28026997

[B16] Harata K. Kubo Y. (2014). Ras GTPase activating protein CoIra1 is involved in infection-related morphogenesis by regulating cAMP and MAPK signaling pathways through CoRas2 in *Colletotrichum orbiculare*. PLoS ONE. 9:e109045. 10.1371/journal.pone.0109045 25275394 PMC4183519

[B17] He P. Wang Y. Wang X. Zhang X. Tian C. (2017). The mitogen-activated protein kinase *CgMK1* governs appressorium formation, melanin synthesis, and plant infection of *Colletotrichum gloeosporioides*. Front. Microbiol. 8:2216. 10.3389/fmicb.2017.02216 29176970 PMC5686099

[B18] Heese A. Hann D. R. Gimenez-Ibanez S. Jones A. Rathjen J. P. (2007). The receptor-like kinase SERK3/BAK1 is a central regulator of innate immunity in plants. Proc. Natl. Acad. Sci. U. S. A. 104, 12217–12222. 10.1073/pnas.0705306104 17626179 PMC1924592

[B19] Hergovich A. Stegert M. R. Schmitz D. Hemmings B. A. (2006). NDR kinases regulate essential cell processes from yeast to humans. Nat. Rev. Mol. Cell Biol. 7, 253–264. 10.1038/nrm1891 16607288

[B20] Huffaker A. Pearce G. Ryan C. A. (2006). An endogenous peptide signal in *Arabidopsis* activates components of the innate immune response. Proc. Natl. Acad. Sci. U. S. A. 103, 10098–10103. 10.1073/pnas.0603727103 16785434 PMC1502512

[B21] Irieda H. Inoue Y. Mori M. Yamada K. Oshikawa Y. Saitoh H.. (2019). Conserved fungal effector suppresses PAMP-triggered immunity by targeting plant immune kinases. Proc. Natl. Acad. Sci. U. S. A. 116, 496–505. 10.1073/pnas.1807297116 30584105 PMC6329965

[B22] Ishihama N. Yamada R. Yoshioka M. Katou S. Yoshioka H. (2011). Phosphorylation of the *Nicotiana benthamiana* WRKY8 transcription factor by MAPK functions in the defense response. Plant Cell. 23, 1153–1170. 10.1105/tpc.110.081794 21386030 PMC3082260

[B23] Jones J. D. Dangl J. L. (2006). The plant immune system. Nature. 444, 323–329. 10.1038/nature05286 17108957

[B24] Kadota Y. Sklenar J. Derbyshire P. Stransfeld L. Asai S. Ntoukakis V.. (2014). Direct regulation of the NADPH oxidase RBOHD by the PRR-associated kinase BIK1 during plant immunity. Mol. Cell. 54, 43–55. 10.1016/j.molcel.2014.02.021 24630626

[B25] Kim Y. K. Kawano T. Li D. Kolattukudy P. E. (2000). A mitogen-activated protein kinase kinase required for induction of cytokinesis and appressorium formation by host signals in the conidia of *Colletotrichum gloeosporioides*. Plant Cell. 12, 1331–1343. 10.1105/tpc.12.8.1331 10948253 PMC149106

[B26] Kodama S. Ishizuka J. Miyashita I. Ishii T. Nishiuchi T. Miyoshi H.. (2017). The morphogenesis-related NDR kinase pathway of *Colletotrichum orbiculare* is required for translating plant surface signals into infection-related morphogenesis and pathogenesis. PLoS Pathog. 13, e1006189. 10.1371/journal.ppat.1006189 28146587 PMC5305266

[B27] Kojima K. Kikuchi T. Takano Y. Oshiro E. Okuno T. (2002). The mitogen-activated protein kinase gene *MAF1* is essential for the early differentiation phase of appressorium formation in *Colletotrichum lagenarium*. Mol. Plant-Microbe Interact. 15, 1268–1276. 10.1094/MPMI.2002.15.12.1268 12481999

[B28] Krol E. Mentzel T. Chinchilla D. Boller T. Felix G. Kemmerling B.. (2010). Perception of the *Arabidopsis* danger signal peptide 1 involves the pattern recognition receptor *At*PEPR1 and its close homologue *At*PEPR2. J. Biol. Chem. 285, 13471–13479. 10.1074/jbc.M109.097394 20200150 PMC2859507

[B29] Kronstad J. W. Hu G. Choi J. (2011). The cAMP/protein kinase A pathway and virulence in *Cryptococcus neoformans*. Mycobiology. 39, 143–150. 10.5941/MYCO.2011.39.3.143 22783095 PMC3385117

[B30] Levin D. E (2005). Cell wall integrity signaling in *Saccharomyces* cerevisiae. Microbiol. Mol. Biol. Rev. 69, 262–291. 10.1128/MMBR.69.2.262-291.2005 15944456 PMC1197416

[B31] Li D. Agrellos O. A. Calderone R. (2010). Histidine kinases keep fungi safe and vigorous. Curr. Opin. Microbiol. 13, 424–430. 10.1016/j.mib.2010.04.007 20542727

[B32] Li L. Li M. Yu L. Zhou Z. Liang X. Liu Z.. (2014). The FLS2-associated kinase BIK1 directly phosphorylates the NADPH oxidase RbohD to control plant immunity. Cell Host Microbe. 15, 329–338. 10.1016/j.chom.2014.02.009 24629339

[B33] Li T. Fan P. Yun Z. Jiang G. Zhang Z. Jiang Y. (2019a). β-Aminobutyric acid priming acquisition and defense response of mango fruit to *Colletotrichum gloeosporioides* infection based on quantitative proteomics. Cells. 8:1029. 10.3390/cells8091029 31487826 PMC6770319

[B34] Li T. Wu Q. Zhu H. Zhou Y. Jiang Y. Gao H.. (2019b). Comparative transcriptomic and metabolic analysis reveals the effect of melatonin on delaying anthracnose incidence upon postharvest banana fruit peel. BMC Plant Biol. 19:289. 10.1186/s12870-019-1855-2 31262259 PMC6604187

[B35] Li Y. He P. Tian C. Wang Y. (2020). *CgHog1* controls the adaptation to both sorbitol and fludioxonil in *Colletotrichum gloeosporioides*. Fungal. Genet. Biol. 135:103289. 10.1016/j.fgb.2019.103289 31704368

[B36] Liang X. Wei T. Cao M. Zhang X. Liu W. Kong Y.. (2019). The MAP kinase CfPMK1 is a key regulator of pathogenesis, development, and stress tolerance of *Colletotrichum fructicola*. Front. Microbiol. 10:1070. 10.3389/fmicb.2019.01070 31164876 PMC6536633

[B37] Liang X. Zhou J. M. (2018). Receptor-like cytoplasmic kinases: central players in plant receptor kinase-mediated signaling. Annu. Rev. Plant Biol. 69, 267–299. 10.1146/annurev-arplant-042817-040540 29719165

[B38] Lu D. Wu S. Gao X. Zhang Y. Shan L. He P. (2010). A receptor-like cytoplasmic kinase, BIK1, associates with a flagellin receptor complex to initiate plant innate immunity. Proc. Natl. Acad. Sci. U. S. A. 107, 496–501. 10.1073/pnas.0909705107 20018686 PMC2806711

[B39] Meng X. Zhang S. (2013). MAPK cascades in plant disease resistance signaling. Annu. Rev. Phytopathol. 51, 245–266. 10.1146/annurev-phyto-082712-102314 23663002

[B40] Miranda V. J. Porto W. F. Fernandes G. D. R. Pogue R. Nolasco D. O. Araujo A. C. G.. (2017). Comparative transcriptomic analysis indicates genes associated with local and systemic resistance to *Colletotrichum graminicola* in maize. Sci. Rep. 7:2483. 10.1038/s41598-017-02298-8 28559543 PMC5449407

[B41] Nogueira G. B. dos Santos L. V. de Queiroz C. B. Correa T. L. R. Menicucci R. P. Bazzolli D. M. S.. (2019). The histidine kinase slnCl1 of *Colletotrichum lindemuthianum* as a pathogenicity factor against *Phaseolus vulgaris* L. Microbiol. Res. 219, 110–122. 10.1016/j.micres.2018.10.005 30642461

[B42] Nühse T. S. Bottrill A. R. Jones A. M. Peck S. C. (2007). Quantitative phosphoproteomic analysis of plasma membrane proteins reveals regulatory mechanisms of plant innate immune responses. Plant J. 51, 931–940. Epub 2007 Jul 25. 10.1111/j.1365-313X.2007.03192.x 17651370 PMC2156193

[B43] Oblessuc P. R. Francisco C. Melotto M. (2015). The *Co-4* locus on chromosome Pv08 contains a unique cluster of 18 *COK-4* genes and is regulated by immune response in common bean. Theor. Appl. Genet. 128, 1193–1208. 10.1007/s00122-015-2500-6 25805316

[B44] O'Connell R. Herbert C. Sreenivasaprasad S. Khatib M. Dumas B. (2004). A novel *Arabidopsis*-*Colletotrichum* pathosystem for the molecular dissection of plant-fungal interactions. Mol. Plant-Microbe Interact. 17, 272–282. 10.1094/MPMI.2004.17.3.272 15000394

[B45] O'Connell R. J. Thon M. R. Hacquard S. myotte S. G. Kleemann J. Torres M. F. (2012). Lifestyle transitions in plant pathogenic *Colletotrichum* fungi deciphered by genome and transcriptome analyses. Nat. Genet. 44, 1060–1065. 10.1038/ng.2372 22885923 PMC9754331

[B46] Pitzschke A. Schikora A. Hirt H. (2009). MAPK cascade signalling networks in plant defence. Curr. Opin. Plant. Biol. 12, 421–426. 10.1016/j.pbi.2009.06.008 19608449

[B47] Priyatno T. P. Abu Bakar F. D. Kamaruddin N. Mahadi N. M. Abdul Murad A. M. (2012). Inactivation of the catalytic subunit of cAMP-dependent protein kinase A causes delayed appressorium formation and reduced pathogenicity of *Colletotrichum gloeosporioides*. Sci. World J. 2012:545784. 10.1100/2012/545784 22666136 PMC3361302

[B48] Ren D. Yang K. Y. Li G. Liu Y. Zhang S. (2006). Activation of Ntf4, a tobacco mitogen-activated protein kinase, during plant defense response and its involvement in hypersensitive response-like cell death. Plant Physiol. 141, 1482–1493. 10.1104/pp.106.080697 16798947 PMC1533962

[B49] Richard M. M. Pflieger S. Sevignac M. Thareau V. Blanchet S. Li Y.. (2014). Fine mapping of *Co-x*, an anthracnose resistance gene to a highly virulent strain of *Colletotrichum lindemuthianum* in common bean. Theor. Appl. Genet. 127, 1653–1666. 10.1007/s00122-014-2328-5 24859268

[B50] Richard M. M. S. Gratias A. Alvarez Diaz J. C. Thareau V. Pflieger S. Meziadi C.. (2021). A common bean truncated CRINKLY4 kinase controls gene-for-gene resistance to the fungus *Colletotrichum lindemuthianum*. J. Exp. Bot. 72, 3569–3581. 10.1093/jxb/erab082 33693665

[B51] Robertson L. S. Fink G. R. (1998). The three yeast A kinases have specific signaling functions in pseudohyphal growth. Proc. Natl. Acad. Sci. U. S. A. 95, 13783–13787. 10.1073/pnas.95.23.13783 9811878 PMC24897

[B52] Rockenbach M. F. Boneti J. I. Cangahuala-Inocente G. C. Gavioli-Nascimento M. C. A. Guerra M. P. (2015). Histological and proteomics analysis of apple defense responses to the development of *Colletotrichum gloeosporioides* on leaves. Physiol. Mol. Plant Pathol. 89, 97–107. 10.1016/j.pmpp.2015.01.003

[B53] Saijo Y. Loo E. P. Yasuda S. (2018). Pattern recognition receptors and signaling in plant-microbe interactions. Plant J. 93, 592–613. 10.1111/tpj.13808 29266555

[B54] Saito H. Posas F. (2012). Response to hyperosmotic stress. Genetics. 192, 289–318. 10.1534/genetics.112.140863 23028184 PMC3454867

[B55] Sakaguchi A. Tsuji G. Kubo Y. (2010). A yeast *STE11* homologue *CoMEKK1* is essential for pathogenesis-related morphogenesis in *Colletotrichum orbiculare*. Mol. Plant. Microbe. Interact. 23, 1563–1572. 10.1094/MPMI-03-10-0051 21039273

[B56] Schmidpeter J. Dahl M. Hofmann J. Koch C. (2017). ChMob2 binds to ChCbk1 and promotes virulence and conidiation of the fungal pathogen *Colletotrichum higginsianum*. BMC Microbiol. 17:22. 10.1186/s12866-017-0932-7 28103800 PMC5248491

[B57] Shan D. Wang C. Zheng X. Hu Z. Zhu Y. Zhao Y.. (2021). MKK4-MPK3-WRKY17-mediated salicylic acid degradation increases susceptibility to Glomerella leaf spot in apple. Plant Physiol. 186, 1202–1219. 10.1093/plphys/kiab108 33693824 PMC8195508

[B58] Shi J. An H. L. Zhang L. Gao Z. Guo X. Q. (2010). *GhMPK7*, a novel multiple stress-responsive cotton group C MAPK gene, has a role in broad spectrum disease resistance and plant development. Plant Mol. Biol. 74, 1–17. 10.1007/s11103-010-9661-0 20602149

[B59] Shi J. Zhang L. An H. Wu C. Guo X. (2011). *GhMPK16*, a novel stress-responsive group D MAPK gene from cotton, is involved in disease resistance and drought sensitivity. BMC Mol. Biol. 12, 22. 10.1186/1471-2199-12-22 21575189 PMC3117701

[B60] Silva L. Moreno H. Correia H. Santana M. F. Queiroz M. (2020). *Colletotrichum*: species complexes, lifestyle, and peculiarities of some sources of genetic variability. Appl. Microbiol. Biotechnol. 104, 1891–1904. 10.1007/s00253-020-10363-y 31932894

[B61] Srideepthi R. Krishna M. S. R. Suneetha P. Krishna R. S. Karthikeyan S. (2020). Genome-wide identification, characterization and expression analysis of non-RD receptor like kinase gene family under *Colletotrichum truncatum* stress conditions in hot pepper. Genetica. 148, 283–296. 10.1007/s10709-020-00104-4 32918190

[B62] Takano Y. Kikuchi T. Kubo Y. Hamer J. E. Mise K. Furusawa I. (2000). The *Colletotrichum lagenarium* MAP kinase gene *CMK1* regulates diverse aspects of fungal pathogenesis. Mol. Plant-Microbe Interact. 13, 374–383. 10.1094/MPMI.2000.13.4.374 10755300

[B63] Takano Y. Komeda K. Kojima K. Okuno T. (2001). Proper regulation of cyclic AMP-dependent protein kinase is required for growth, conidiation, and appressorium function in the anthracnose fungus *Colletotrichum lagenarium*. Mol. Plant-Microbe Interact. 14, 1149–1157. 10.1094/MPMI.2001.14.10.1149 11605954

[B64] Tanaka S. Ishihama N. Yoshioka H. Huser A. O'Connell R. Tsuji G.. (2009). The *Colletotrichum orbiculare SSD1* mutant enhances Nicotiana benthamiana basal resistance by activating a mitogen-activated protein kinase pathway. Plant Cell. 21, 2517–2526. 10.1105/tpc.109.068023 19706796 PMC2751964

[B65] Tang D. Wang G. Zhou J. M. (2017). Receptor kinases in plant-pathogen interactions: more than pattern recognition. Plant Cell. 29, 618–637. 10.1105/tpc.16.00891 28302675 PMC5435430

[B66] Truesdell G. M. Jones C. Holt T. Henderson G. Dickman M. B. (1999). A Ras protein from a phytopathogenic fungus causes defects in hyphal growth polarity, and induces tumors in mice. Mol. Gen. Genet. 262, 46–54. 10.1007/s004380051058 10503535

[B67] Turra D. Segorbe D. Di Pietro A. (2014). Protein kinases in plant-pathogenic fungi: conserved regulators of infection. Annu. Rev. Phytopathol. 52, 267–288. 10.1146/annurev-phyto-102313-050143 25090477

[B68] Wang X. Lu D. Tian C. (2021). Mitogen-activated protein kinase cascade CgSte50-Ste11-Ste7-Mk1 regulates infection-related morphogenesis in the poplar anthracnose fungus *Colletotrichum gloeosporioides*. Microbiol. Res. 248:126748. 10.1016/j.micres.2021.126748 33752111

[B69] Wei W. Xiong Y. Zhu W. Wang N. Yang G. Peng F. (2016). *Colletotrichum higginsianum* mitogen-activated protein kinase ChMK1: role in growth, cell wall integrity, colony melanization, and pathogenicity. Front. Microbiol. 7:1212. 10.3389/fmicb.2016.01212 27536296 PMC4971432

[B70] Widmann C. Gibson S. Jarpe M. B. Johnson G. L. (1999). Mitogen-activated protein kinase: conservation of a three-kinase module from yeast to human. Physiol. Rev. 79, 143–180. 10.1152/physrev.1999.79.1.143 9922370

[B71] Xiong Q. Xu J. Zhao Y. Wang K. (2015). *CtPMK1*, a mitogen-activated-protein kinase gene, is required for conidiation, appressorium formation, and pathogenicity of *Colletotrichum truncatumon* soybean. Ann. Appl. Biol. 167, 63–74. 10.1111/aab.12209

[B72] Xu J. R (2000). MAP kinases in fungal pathogens. Fungal Genet. Biol. 31, 137–152. 10.1006/fgbi.2000.1237 11273677

[B73] Xu X. Wang Y. Tian C. Liang Y. (2016). The *Colletotrichum gloeosporioides* RhoB regulates cAMP and stress response pathways and is required for pathogenesis. Fungal. Genet. Biol. 96, 12–24. 10.1016/j.fgb.2016.09.002 27670809

[B74] Yamada K. Yamashita-Yamada M. Hirase T. Fujiwara T. Tsuda K. Hiruma K.. (2016). Danger peptide receptor signaling in plants ensures basal immunity upon pathogen-induced depletion of BAK1. EMBO J. 35, 46–61. 10.15252/embj.201591807 26574534 PMC4718002

[B75] Yamauchi J. Takayanagi N. Komeda K. Takano Y. Okuno T. (2004). cAMP-pKA signaling regulates multiple steps of fungal infection cooperatively with Cmk1 MAP kinase in *Colletotrichum lagenarium*. Mol. Plant-Microbe Interact. 17, 1355–1365. 10.1094/MPMI.2004.17.12.1355 15597741

[B76] Yan Y. Tang J. Yuan Q. Gu Q. Liu H. Huang J.. (2020). *ChCDC25* regulates infection-related morphogenesis and pathogenicity of the crucifer anthracnose fungus *Colletotrichum higginsianum*. Front. Microbiol. 11:763. 10.3389/fmicb.2020.00763 32457707 PMC7227425

[B77] Yang Z. Dickman M. B. (1999). *Colletotrichum trifolii* mutants disrupted in the catalytic subunit of cAMP-dependent protein kinase are nonpathogenic. Mol. Plant- Microbe Interact. 12, 430–439. 10.1094/MPMI.1999.12.5.430 10226376

[B78] Yip Delormel T. Boudsocq M. (2019). Properties and functions of calcium-dependent protein kinases and their relatives in *Arabidopsis thaliana*. New Phytol. 224:585–604. 10.1111/nph.16088 31369160

[B79] Yong H. Y. Bakar F. D. Illias R. M. Mahadi N. M. Murad A. M. (2013). *Cgl-SLT2* is required for appressorium formation, sporulation and pathogenicity in *Colletotrichum gloeosporioides*. Braz. J. Microbiol. 44, 1241–1250. 10.1590/S1517-83822013000400031 24688518 PMC3958194

[B80] Yu H. Yan J. Qiu J. Wang S. Xin Y. Tong J.. (2019). Differential phosphoproteomic analysis of strawberry in response to *Colletotrichum gloeosporioides* (in chinese). ZHEJIANG UNIV. 4, 418–425.

[B81] Yuan Q. Chen M. Yan Y. Gu Q. Huang J. Zheng L. (2016). *ChSte7* is required for vegetative growth and various plant infection processes in *Colletotrichum higginsianum*. Biomed Res. Int. 2016:7496569. 10.1155/2016/7496569 27563675 PMC4987456

[B82] Zhang J. Li W. Xiang T. Liu Z. Laluk K. Ding X.. (2010). Receptor-like cytoplasmic kinases integrate signaling from multiple plant immune receptors and are targeted by a *Pseudomonas syringae* effector. Cell Host Microbe 7, 290–301. 10.1016/j.chom.2010.03.007 20413097

[B83] Zhang M. Su J. Zhang Y. Xu J. Zhang S. (2018). Conveying endogenous and exogenous signals: MAPK cascades in plant growth and defense. Curr. Opin. Plant Biol. 45, 1–10. 10.1016/j.pbi.2018.04.012 29753266

[B84] Zhu W. Zhou M. Xiong Z. Peng F. Wei W. (2017). The cAMP-PKA signaling pathway regulates pathogenicity, hyphal growth, appressorial formation, conidiation, and stress tolerance in *Colletotrichum higginsianum*. Front. Microbiol. 8:1416. 10.3389/fmicb.2017.01416 28791004 PMC5524780

[B85] Zipfel C. Kunze G. Chinchilla D. Aniard A. C. Jones J. Boller T.. (2006). Perception of the bacterial PAMP EF-Tu by the receptor EFR restricts *Agrobacterium*-mediated transformation. Cell. 125, 749–760. 10.1016/j.cell.2006.03.037 16713565

[B86] Zipfel C. Robatzek S. Navarro L. Oakeley E. J. Jones J. Felix G.. (2004). Bacterial disease resistance in *Arabidopsis* through flagellin perception. Nature. 428, 764–767. 10.1038/nature02485 15085136

